# Visual and Patient-Reported Outcomes After Bilateral Implantation of Two Enhanced Monofocal IOLs

**DOI:** 10.3390/jcm15103904

**Published:** 2026-05-19

**Authors:** Rosa Giglio, Serena Milan, Riccardo Leonelli, Elena Verdimonti, Alberto Grotto, Marianna Presotto, Giulia Soccio, Marco Zeppieri, Gianluca Turco, Daniele Tognetto

**Affiliations:** 1 Department of Medical, Surgical Sciences and Health, University of Trieste, 34129 Trieste, Italy; giglio.rosam@gmail.com (R.G.); leonelliriccardo96@gmail.com (R.L.); dr.albertogrotto@gmail.com (A.G.); presoctomarianna@gmail.com (M.P.); giuliasoccio@gmail.com (G.S.); gturco@units.it (G.T.); tognetto@units.it (D.T.); 2 Department of Ophthalmology, University Hospital of Udine, 33100 Udine, Italy

**Keywords:** cataract surgery, enhanced monofocal intraocular lens, intermediate visual acuity

## Abstract

**Background/Objectives:** Enhanced monofocal intraocular lenses (IOLs) aim to extend functional vision into the intermediate range while preserving the distance visual quality of standard monofocal aspheric lenses. This study compared the clinical and patient-reported outcomes of two enhanced monofocal IOLs, TECNIS Eyhance™ and Evolux™, and characterised the anterior surface profile of the Evolux™ optic. **Methods:** This single-centre, retrospective comparative case series included consecutive patients who received bilateral implantation of either Evolux™ or TECNIS Eyhance™ between February and July 2023. Primary outcomes were monocular uncorrected and distance-corrected intermediate visual acuity (UIVA, DCIVA). Secondary outcomes included monocular and binocular distance and near visual acuity, binocular intermediate visual acuity, spherical equivalent and patient-reported outcomes assessed using the Revised Heidelberg Daily Task Evaluation (DATE) questionnaire. Equivalence testing, Welch’s *t*-test, and covariate-adjusted ANCOVA were performed. Anterior surface profilometry of the Evolux™ IOL was conducted using an optical profilometer. **Results:** A total of 44 patients were included, 14 in the Evolux™ group and 30 in the TECNIS Eyhance™ group. Monocular and binocular UIVA and DCIVA were statistically equivalent between groups (TOST *p* = 0.028, 0.016, 0.008, and 0.005, respectively). Monocular and binocular distance outcomes were likewise equivalent. Binocular distance-corrected near visual acuity was significantly better in the Evolux™ group (0.164 ± 0.084 vs. 0.233 ± 0.112 logMAR; *p* = 0.030; Cohen’s d = 0.661), without a corresponding monocular difference. This isolated finding should be interpreted cautiously given the exploratory, multiple-outcome analysis and because it did not retain statistical significance after covariate adjustment for baseline biometric imbalances. **Conclusions:** In this exploratory study, both IOLs showed no statistically significant differences in intermediate and distance visual outcomes at one month after second eye surgery. The unadjusted binocular near vision finding for Evolux™, which did not retain significance after covariate adjustment, warrants further investigation in prospective, adequately powered, biometrically balanced studies.

## 1. Introduction

Cataract surgery has evolved beyond the simple restoration of visual acuity. Today, patients increasingly expect to reduce or eliminate their dependence on spectacles for everyday tasks, and the concept of functional vision has emerged as a central goal in modern ophthalmic practice [1,2,3]. A critical component of functional vision is intermediate distance visual acuity, which is essential for many routine tasks such as working on a computer, cooking, reading product labels, and engaging in social interactions. Conventional monofocal intraocular lenses (IOLs), which remain the most widely used option in cataract surgery, are effective in restoring uncorrected visual acuity at a single focal point, usually far. However, they do not adequately address visual demands at intermediate and near distances. Consequently, patients frequently require spectacles for tasks that fall within these ranges. Although advanced IOL technologies, such as multifocal and extended depth of focus (EDOF) lenses, have been developed to reduce dependence on spectacles by providing a broader range of vision, these lenses are not without limitations. Specifically, they have often been associated with undesirable photic phenomena such as halos and glare and might compromise distance image quality and contrast sensitivity compared to monofocal IOLs [4,5]. In recent years, there has been an increase in the availability of IOLs designed to deliver not only excellent distance vision but also improved intermediate visual acuity. Innovative optical technologies have been integrated into monofocal IOL designs, aiming to preserve the high-quality distance vision of standard monofocal IOLs while enhancing performance at intermediate distances, without introducing dysphotopsias commonly associated with multifocal or diffractive lenses [4,6,7]. In 2019, the TECNIS Eyhance™ was the first of a new generation of monofocal IOLs engineered to provide superior intermediate vision compared to conventional aspheric monofocal lenses, while maintaining comparable distance visual acuity [8]. Three years later, the Evolux™, a hydrophobic monofocal IOL, entered the market. Designed with a non-diffractive profile, Evolux™ aims to deliver the same level of visual quality at distance as standard monofocal IOLs, along with enhanced intermediate vision. The primary objective of the present study was to compare the clinical and patient-reported outcomes of the TECNIS Eyhance™ and Evolux™ IOLs, with particular attention to functional vision at distance and intermediate ranges. The secondary objective was to characterise the anterior surface profile of the Evolux™ IOL. The surface profile of the TECNIS Eyhance™ IOL has previously been described in the literature [9]. In the present study, profilometry was used to provide a structural description of the Evolux™ optic [10,11].

## 2. Materials and Methods

The study protocol adhered to the tenets of the Declaration of Helsinki and was approved by the Ethics Committee of the University of Trieste (Italy). The nature and the purpose of the investigation were fully explained, and written informed consent was obtained from all participants. This was a single-centre, retrospective comparative case series. Consecutive patients undergoing bilateral cataract extraction at the University Eye Clinic of Trieste between February and July 2023 were enrolled. Each patient received bilateral implantation of the same IOL model, either Evolux™ or TECNIS Eyhance™.

Group allocation was based on the IOL implanted, and patients were not randomized. The choice of IOL was determined by the operating surgeon (DT) based on clinical judgement and institutional lens availability during the enrolment period. The Evolux™ lens was introduced into routine clinical practice at our centre later within the study window, which accounts for the smaller cohort size in that group. This non-randomized allocation constitutes a potential source of selection bias and should be considered when interpreting all between-group comparisons. Exclusion criteria included anterior segment pathology that could have a significant impact on outcomes (e.g., chronic uveitis, iritis, corneal dystrophy, keratoconus), axial length ≤20 mm or ≥27 mm, corneal astigmatism higher than 1.0 D (absolute value), diabetic retinopathy, uncontrolled glaucoma and/or intraocular pressure (IOP) >24 mmHg, active ocular or systemic infection, traumatic cataract, pseudoexfoliation syndrome, pupillary abnormalities including aniridia and/or pupillary diameter in mesopic conditions in distance vision ≤2.5 mm and ≥6 mm, microphthalmia, amblyopia, degenerative visual disorders (e.g., macular degeneration, optic nerve atrophy or retinal disorders), previous intraocular and corneal surgery, systemic or ocular pharmacotherapy which could impact the visual acuity and/or cause floppy iris syndrome and/or insufficient dilation according to the investigator’s opinion, strabismus, nystagmus, pregnancy or lactation period for female patients. All patients underwent a comprehensive preoperative ophthalmological examination including: medical history recording, measurement of monocular uncorrected distance visual acuity (UDVA) (at 4 m) and corrected distance visual acuity (CDVA), anterior segment slit lamp examination, Goldmann applanation tonometry, fundus oculi examination after dilatation with Tropicamide 1% eye drops, macular optical coherence tomography (Spectralis HRA + OCT; Heidelberg Engineering, Heidelberg, Germany), bilateral optical biometry (IOLMaster700; Carl Zeiss Meditec AG, Jena, Germany), autorefractometry (OPD Scan III; Nidek Co, Gamagori, Japan), corneal topography and pupillometry (Scheimpflug camera—Sirius; C.S.O. Srl, Florence, Italy). Data acquired with IOLMaster700 were used for IOL power calculation. The IOL powers were calculated using the Barrett Universal II formula. The dioptric power with the expected refractive target closest to emmetropia was chosen. Evolux™ (model 1110ACH, SIFI, Catania, Italy) and TECNIS Eyhance™ (model ICB00, Johnson & Johnson Vision Care Inc., Jacksonville, FL, USA) are two 1-piece biconvex UV-light filtering acrylic foldable posterior chamber IOLs designed for placement in the capsular bag, with an overall length of 13 mm and a 6.0 mm optic diameter. They were launched on the market as enhanced monofocal IOLs, both aiming at improving intermediate vision while providing distance vision comparable to a standard monofocal IOL. The TECNIS Eyhance™ has a refractive optical design with a higher-order aspheric anterior surface that creates a continuous power profile. Its power increases continuously from the periphery to the centre of the lens [12,13,14,15]. Evolux™ IOL has a refractive optical profile based on a patented wavefront engineering technology. Its anterior surface features concentric zones in the central 4.5 mm area, characterised by spherical aberrations of opposite signs, while a standard aspheric monofocal profile is maintained at the periphery [16,17]. All procedures were performed by a single experienced surgeon (DT). Standard phacoemulsification through a 2.4 mm clear corneal incision, followed by IOL insertion in the capsular bag under topical anaesthesia, was performed. Anterior capsulotomies were obtained as a continuous, curvilinear capsulorhexis of 5.0 to 5.5 mm diameter by manual rhexis. As per our routine clinical practice postoperative assessments were performed 30 ± 10 days after the second eye surgery. The mean interval between first-eye and second-eye surgery was 30 ± 10 days. The primary outcomes were monocular uncorrected intermediate visual acuity (UIVA) and monocular distance-corrected intermediate visual acuity (DCIVA). Secondary outcomes included postoperative spherical equivalent (SE), uncorrected distance visual acuity (UDVA), corrected distance visual acuity (CDVA), distance-corrected near visual acuity (DCNVA), corrected near visual acuity (CNVA) and near vision correction (NVC). Early Treatment Diabetic Retinopathy Study (ETDRS) acuity charts were used to measure pre- and postoperative visual acuities. Distance Visual Acuity was obtained with a 4 m ETDRS board illumination cabinet at high contrast (96%) with an 85 cd/m2 lamp filter tube (Precision Vision, La Salle, PA, USA), intermediate visual acuity was obtained with a 66 cm ETDRS printed chart (Precision Vision), and near visual acuity was obtained with a 40 cm ETDRS printed chart (Precision Vision). Binocular corrected distance defocus curves were obtained. The defocus curve was obtained by sequentially adding trial lenses to the best distance correction in 0.50 D steps, across a range from +1.00 to −4.00 D. At each step, visual acuity was assessed using a high-contrast (100%) ETDRS chart at a test distance of 4 m. Pupillometry was performed under scotopic, mesopic, and photopic conditions using the Scheimpflug Camera. Patient satisfaction and daily task performance were assessed using the Revised Heidelberg Daily Task Evaluation (DATE) questionnaire [18].

A structural analysis of the anterior surface of the Evolux™ IOL was also carried out by using an optical profilometer (Sensofar S Neox microscope; Sensofar Tech S.L, Terrassa, Spain). An IOL power of +25 D was used for the purpose. A magnification equal to 50× was used to analyse the IOL front surface in interferometric mode. A best-fit spherical surface was subtracted from the raw profile to obtain the two-dimensional representation of the anterior surface profile of the Evolux™ IOL, as previously described [9].

All analyses were performed in Python 3.12. Given the retrospective design, no a priori sample size calculation was performed. All eligible eyes during the study period were included. Because both eyes of each patient underwent bilateral implantation of the same IOL model, continuous eye-level variables were averaged within patient before between-group comparison to preserve statistical independence at the patient level. Questionnaire data are inherently patient-level and were not averaged. For continuous outcomes, we report group means ± standard deviation (SD) and the between-group mean difference (TECNIS Eyhance™-Evolux™) with 95% confidence intervals (CIs) based on Welch’s *t*-test, which does not assume equal variances. Cohen’s d was calculated as a standardized effect size (|d| ≈ 0.2 small, ≈0.5 medium, ≈0.8 large). To assess equivalence, the two one-sided tests (TOST) at α = 0.05 were performed. Pre-specified equivalence margins were ±0.1 logMAR for all visual acuity outcomes, ±0.25 D for spherical equivalent, and ±0.5 D for near vision correction. Equivalence was concluded when the TOST *p*-value fell below 0.05, indicating that the observed difference was unlikely to exceed the clinically meaningful margin in either direction. Given the retrospective design, the absence of an a priori sample size calculation, and the small and imbalanced cohort, these equivalence analyses should be regarded as exploratory endpoint-specific procedures and must not be interpreted as confirmatory demonstrations of clinical equivalence. To assess whether baseline imbalances in anterior chamber depth (ACD) and IOL power influenced postoperative outcomes, we fitted separate one-covariate ANCOVA models for each monocular and binocular visual acuity outcome and for spherical equivalent: one model adjusted for ACD and one model adjusted for IOL power. For both monocular and binocular models, covariates represent patient-level means (average of right and left eye). Given the sample size and collinearity concerns, these covariates were not entered simultaneously. These models should be considered exploratory sensitivity analyses only and are not intended as confirmatory adjustments; residual confounding attributable to unmeasured variables and to the limited sample size cannot be excluded. For the DATE questionnaire, for binary items (Yes/No), Fisher’s exact test was used, and results are expressed as risk differences (RD) in percentage points. For three-category items (Yes/Partly/No) and for ordinal frequency items (Never/Rarely/Sometimes/Frequently/Always), the Mann–Whitney U (MWU) test was applied, treating response categories as ordered scores. Fisher’s exact test was additionally applied to three-category items (Yes vs. Partly + No collapsed). No correction for multiple comparisons was applied, given the exploratory nature of this analysis; results should be interpreted accordingly.

During the preparation of this manuscript, the authors used Claude (Anthropic, claude.ai) to assist with statistical code writing, verification of results, language editing and manuscript review. The authors have reviewed and edited the output and take full responsibility for the content of this publication.

## 3. Results

A total of 88 eyes of 44 patients were included: 30 patients (60 eyes) in the TECNIS Eyhance™ group and 14 patients (28 eyes) in the Evolux™ group. For all monocular outcomes, continuous variables were averaged between right and left eye prior to statistical comparison to preserve statistical independence at the patient level; all 44 patients (88 eyes) contributed to each mean. Binocular outcomes were recorded as a single per-patient measurement and required no averaging. Demographic and biometric data are summarized in Table 1.

IOL power was significantly higher in the TECNIS Eyhance™ group than in the Evolux™ group (22.16 ± 1.77 vs. 20.59 ± 2.13 D; mean difference 1.57 D, 95% CI 0.21 to 2.93; *p* = 0.026), whereas ACD showed a medium effect size between-group difference that did not reach statistical significance at the patient level (3.15 ± 0.39 vs. 2.92 ± 0.33 mm; mean difference 0.23 mm, 95% CI −0.01 to 0.46; *p* = 0.056). Monocular postoperative outcomes are shown in Table 2.

No statistically significant between-group differences were observed for any monocular visual acuity outcome or for spherical equivalent. The two largest monocular effect sizes by absolute magnitude were comparable and were observed for corrected distance visual acuity (CDVA; d = 0.604) and distance-corrected near visual acuity (DCNVA; d = 0.601). The DCNVA difference favored TECNIS Eyhance™ numerically (0.255 ± 0.118 vs. 0.325 ± 0.112 logMAR) but did not reach statistical significance (*p* = 0.069), while the CDVA difference was similarly non-significant (*p* = 0.122). Equivalence testing showed that mono-UDVA, mono-CDVA, mono-UIVA, mono-DCIVA, mono-CNVA, and NVC met the prespecified equivalence criteria, whereas equivalence was not demonstrated for monocular DCNVA or spherical equivalent. Binocular outcomes are shown in Table 3.

Binocular DCNVA was significantly better in the Evolux™ group (0.164 ± 0.084 vs. 0.233 ± 0.112 logMAR; difference 0.069 logMAR, 95% CI 0.007 to 0.131; *p* = 0.030; Cohen’s d = 0.661); equivalence was not demonstrated for this outcome (TOST *p* = 0.158). All other binocular outcomes did not differ significantly between groups. Equivalence was demonstrated for bino-UDVA (TOST *p* = 0.031), bino-CDVA (TOST *p* = 0.021), bino-UIVA (TOST *p* = 0.008), bino-DCIVA (TOST *p* = 0.005), and bino-CNVA (TOST *p* < 0.001), but not for bino-DCNVA. After adjustment for ACD, monocular CDVA showed a statistically significant adjusted group difference (adjusted difference 0.043 logMAR; *p* = 0.027), whereas all other monocular adjusted models were non-significant. Adjustment for IOL power did not yield statistically significant group differences for any monocular outcome; the DCNVA model approached the threshold (*p* = 0.057), consistent with the unadjusted trend. Results are presented in Table 4.

To extend the covariate adjustment to binocular outcomes, separate one-covariate ANCOVA models were fitted for each binocular visual acuity outcome, adjusting for ACD and IOL power in turn, following the same approach applied to monocular outcomes. Results are presented in Table 5. After adjustment for ACD, binocular UDVA showed a statistically significant group difference (adjusted difference +0.064 logMAR; *p* = 0.015), indicating that TECNIS Eyhance™ was associated with worse binocular uncorrected distance acuity once ACD was accounted for. After adjustment for IOL power, binocular CDVA reached statistical significance (adjusted difference −0.045 logMAR; *p* = 0.047), confirming that TECNIS Eyhance™ had better binocular corrected distance acuity after controlling for the power imbalance between groups, consistent with the large unadjusted effect size (d = −0.771). Critically, binocular DCNVA, the only unadjusted significant finding, did not retain statistical significance after adjustment for either ACD (adjusted difference +0.067 logMAR; *p* = 0.071) or IOL power (adjusted difference +0.063 logMAR; *p* = 0.096). All remaining binocular outcomes were non-significant after either adjustment. These models should be considered exploratory sensitivity analyses only and are not intended as confirmatory adjustments; residual confounding cannot be excluded.

**Figure 1:**
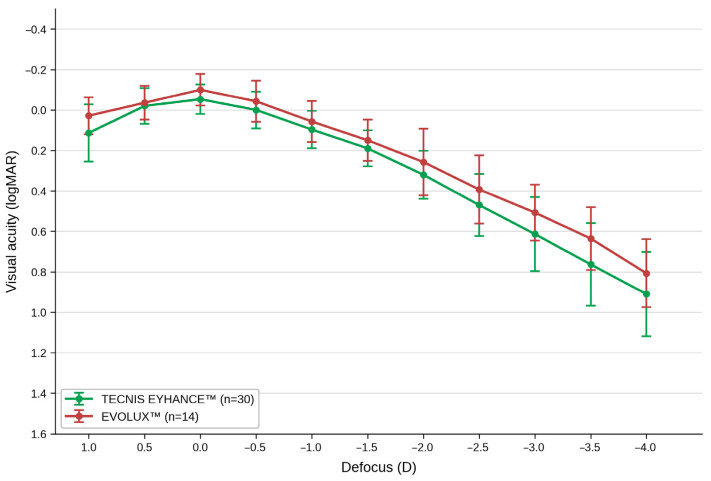
Binocular corrected distance defocus curves. Mean visual acuity (logMAR) ± standard deviation is plotted as a function of defocus (diopters) for the TECNIS Eyhance™ group (*n* = 30, green) and the Evolux™ group (*n* = 14, red). The defocus curve was obtained by sequentially adding trial lenses to the best distance correction in 0.50 D steps, from +1.00 D to −4.00 D. Visual acuity was assessed at each step using a high-contrast (100%) ETDRS chart at 4 m. Lower logMAR values indicate better visual acuity.

Mean binocular defocus curves are shown in Figure 1.

Questionnaire responses were available for 19/30 (63%) TECNIS Eyhance™ patients and 13/14 (93%) Evolux™ patients. Results are presented in Table 6 and Table 7.

**Figure 2:**
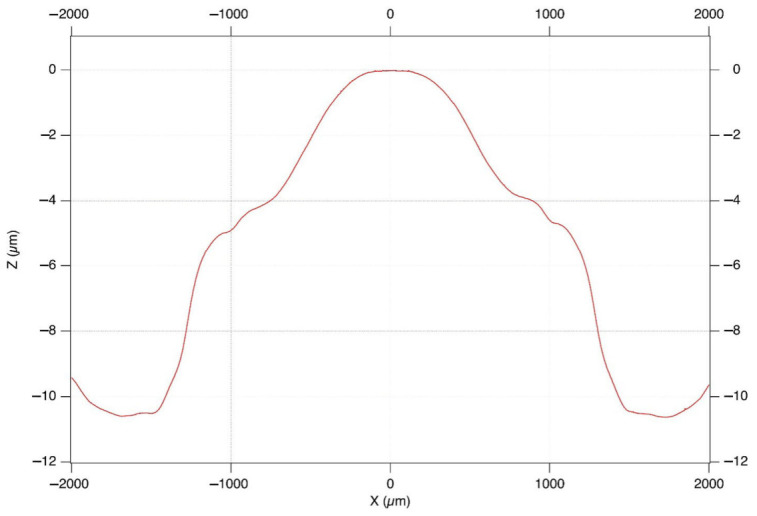
The 2D representation shows the anterior surface profile of Evolux IOL in the 4 central millimetres. The *x*-axis represents the distance from the IOL centre (0 µm).

Satisfaction and willingness to recommend were high in both groups. Overall satisfaction (“Happy with outcome?”) was 73.7% (TECNIS Eyhance™) vs. 84.6% (Evolux™; RD = −10.9 pp; Fisher *p* = 0.671; MWU *p* = 0.575). Willingness to select the same lens again was comparable (89.5% vs. 84.6%; Fisher *p* = 1.000; MWU *p* = 0.713), as was willingness to recommend the surgery (89.5% vs. 84.6%; Fisher *p* = 1.000). No statistically significant between-group differences were found for any satisfaction item. For glass-free functional activities, no significant differences were found for any item. Most tasks with high visual demands, such as watching television (84.2% vs. 100%), driving in daytime and at night (all drivers answered “Yes” in both groups), and working at home or in the garden (89.5% vs. 76.9%), showed high affirmative response rates in both groups. The only notable numerical difference was in shopping at the supermarket without glasses (52.6% TECNIS Eyhance™ vs. 30.8% Evolux™; RD = +21.9 pp; Fisher *p* = 0.289; MWU *p* = 0.196), which did not reach significance. Near-dependent tasks, such as reading newspapers and books, were poorly performed without glasses in both groups (most patients responded “No”), with no between-group difference. Dysphotopsia symptoms were infrequent in both groups, with no statistically significant between-group differences. Reading glasses appeared to be used more often in the TECNIS Eyhance™ group than in the Evolux™ group, but this difference did not reach statistical significance (MWU *p* = 0.088) and should be interpreted cautiously. Distance and intermediate glasses use did not differ significantly between groups. Figure 2 shows the anterior surface profile of Evolux IOL +25.0 D obtained with the optical profilometer.

After the removal of the best-fit spherical surface from the raw measurement, the radial profile showed a high-order aspherical design. Specifically, Evolux™ showed a central steepening and a symmetric gradual curvature variation due to the IOL optical design. In greater detail, a ΔZ variation equal to ~5 µm was recorded in the central 2 mm diameter zone, which is designed to provide intermediate vision. A ΔZ variation equal to ~4 µm was measured in the annular zone between 2 mm and 3 mm optical diameters, which corresponds to a gradual transition zone between the central zone, specific for intermediate vision, and the monofocal peripheral part, which provides far vision. The last portion of the IOL active zone, between 3 mm and 4 mm optical diameters, showed a plateau: a ΔZ variation equal to ~1 µm connected this portion to the aspheric monofocal peripheral zone. Overall, a ΔZ variation equal to ~ 10 µm was observed between the IOL centre and the end of the 4.5 mm active zone.

## 4. Discussion

Enhanced monofocal IOLs represent a design evolution within the monofocal category, aiming to extend functional vision into the intermediate range while preserving the distance visual quality and optical tolerability associated with standard monofocal aspheric lenses. In the present study, we compared the clinical and patient-reported outcomes of two such lenses, TECNIS Eyhance™ and Evolux™, in a consecutive patient cohort undergoing bilateral cataract surgery. The principal finding regarding intermediate vision was that both monocular and binocular outcomes were statistically equivalent between the two lenses. Monocular UIVA and DCIVA showed very small absolute differences (−0.034 and −0.032 logMAR, respectively) with effect sizes in the small range (d = −0.302 and −0.268), and both met the prespecified equivalence criteria (TOST *p* = 0.028 and 0.016, respectively). Binocular UIVA and DCIVA were likewise equivalent (TOST *p* = 0.008 and 0.005, respectively), with negligible effect sizes (d = −0.074 and −0.192). The monocular and binocular equivalence findings at intermediate distance are of descriptive interest and are consistent with both lenses fulfilling their stated design objective within the constraints of this exploratory study. These results are consistent with those reported for both IOLs in comparisons with standard monofocal IOLs, which have demonstrated improved intermediate visual acuity [13,14,15,16,19,20,21,22,23]. However, given the underpowered and non-randomized design, these findings cannot be interpreted as a definitive demonstration of clinical equivalence, and a meaningful performance difference at intermediate distance cannot be formally excluded with the present study.

Monocular distance outcomes were equivalent between groups. Both mono-UDVA and mono-CDVA met the prespecified equivalence criteria (TOST *p* = 0.005 and 0.003, respectively), and neither reached statistical significance on direct comparison (*p* = 0.455 and 0.122, respectively). At the binocular level, UDVA was also equivalent (TOST *p* = 0.031). Binocular CDVA showed a large effect size (d = −0.771) with a *p*-value approaching the conventional threshold (*p* = 0.053), and equivalence was demonstrated (TOST *p* = 0.021). While the between-group difference did not reach statistical significance, the magnitude of this effect (d = 0.771) indicates that a clinically meaningful difference in binocular corrected distance acuity cannot be excluded. In a study of this size, the absence of statistical significance is likely to reflect insufficient power rather than a true null effect, and this finding should accordingly be regarded as requiring verification in a prospective, adequately powered study rather than as evidence of clinical equivalence.

Spherical equivalent was numerically closer to emmetropia in the TECNIS Eyhance™ group (0.032 ± 0.275 D vs. −0.107 ± 0.258 D), but this difference did not reach statistical significance (*p* = 0.113), and equivalence testing did not confirm equivalence at the prespecified ±0.25 D margin (TOST *p* = 0.103). The trend toward a slightly myopic shift in the Evolux™ group may reflect the distinct anterior surface geometry of this lens and its interaction with the Barrett Universal II formula, which was developed primarily on standard aspheric designs. Dedicated optimization of the A-constant or formula selection for new types of aspheric design could further improve refractive predictability and merits prospective evaluation [10]. Baseline biometric differences between groups require careful consideration. IOL power was significantly higher in the TECNIS Eyhance™ group (22.16 ± 1.77 vs. 20.59 ± 2.13 D; *p* = 0.026), reflecting the non-randomized design and the consecutive enrolment of patients. Anterior chamber depth showed a medium effect size difference (d = 0.60) that narrowly missed significance (*p* = 0.056). These imbalances prompted an ANCOVA adjustment. After adjustment for ACD, monocular CDVA reached statistical significance (adjusted difference 0.043 logMAR; *p* = 0.027), suggesting that the unadjusted CDVA comparison may have been partially confounded by differences in effective lens position. ACD is a critical determinant of effective lens position, and even when modern formulae such as Barrett Universal II are applied, residual prediction variability attributable to ACD can influence postoperative refraction and, consequently, corrected distance acuity [24]. By contrast, adjustment for IOL power did not yield significance for any monocular outcome. However, at the binocular level, CDVA reached significance after IOL power adjustment (adjusted difference −0.045 logMAR; *p* = 0.047; Table 5), suggesting that the IOL power imbalance may have contributed to the binocular distance acuity signal, consistent with the large effect size (d = −0.771). After adjustment for ACD, binocular UDVA also reached significance (adjusted difference +0.064 logMAR; *p* = 0.015), indicating that TECNIS Eyhance™ was associated with worse binocular uncorrected distance acuity once ACD was accounted for. Both covariate-adjusted binocular distance findings should be regarded as exploratory signals requiring prospective verification, and the binocular CDVA result is consistent with the earlier interpretation that its unadjusted non-significance likely reflects insufficient power rather than a true null effect. It should be noted that TOST equivalence and ANCOVA significance for binocular UDVA are not contradictory: TOST tests whether the difference exceeds the prespecified ±0.1 logMAR clinical margin, which it does not (hence equivalent), while ANCOVA tests whether the difference is statistically non-zero after removing the confounding effect of ACD, which it is. Both conclusions can therefore hold simultaneously. These findings underscore the importance of covariate-adjusted analyses in non-randomized comparative series, and the ACD-adjusted monocular CDVA result should be interpreted as a signal warranting verification in a prospective design rather than a definitive conclusion. A comparison by Spagnuolo et al. reported a broad distance advantage for Evolux™ over TECNIS Eyhance™ across all tested distances [25], which contrasts with our equivalence findings. Differences in study design, patient selection, follow-up duration, and statistical framework may account for this discrepancy, and direct reconciliation between studies is not straightforward. At the monocular level, DCNVA showed a numerical trend favoring TECNIS Eyhance™ (0.255 ± 0.118 vs. 0.325 ± 0.112 logMAR) that did not reach statistical significance (*p* = 0.069) and for which equivalence was not demonstrated (TOST *p* = 0.212). The moderate effect size observed for this outcome (d = 0.601) indicates that the absence of statistical significance is likely attributable to limited power, and a clinically meaningful monocular near vision difference between the two lenses cannot be excluded based on the present data. By contrast, monocular CNVA was statistically equivalent between groups (TOST *p* < 0.001, d = 0.266), and NVC was similarly equivalent (TOST *p* = 0.003), confirming that the groups were matched for near refractive correction. Cano-Ortiz et al. reported monocular DCNVA values of 0.36 ± 0.16 logMAR following Evolux™ implantation [23], which is numerically comparable to the 0.325 ± 0.112 logMAR observed in the present series. The most notable near vision finding emerged at the binocular level. Binocular DCNVA was significantly better in the Evolux™ group (0.164 ± 0.084 vs. 0.233 ± 0.112 logMAR; difference 0.069 logMAR, 95% CI 0.007 to 0.131; *p* = 0.030; d = 0.661), a medium-to-large effect size, whereas no significant monocular near vision difference was observed. Binocular CNVA was equivalent between groups (TOST *p* < 0.001), indicating that the binocular finding was specific to distance-corrected near acuity rather than a general near vision superiority. This binocular and monocular discrepancy is unlikely to reflect a systematic refractive difference, given that NVC was equivalent and monocular DCNVA showed only a non-significant numerical trend. Given the small cohort size, the exact mechanism remains unclear; however, one possible explanation is a lens-specific binocular summation effect. It is plausible that the optical profile of the Evolux™ lens, characterised by concentric zones with alternating spherical aberration signs across the central optical area, may interact differently with binocular integration than the continuous power profile of the TECNIS Eyhance™. Alternatively, residual interocular differences in the aberration profile between fellow eyes might also contribute differently to binocular summation depending on lens design. However, these interpretations must be regarded as speculative, as the covariate-adjusted ANCOVA results substantially weaken the case for a genuine lens-specific effect. Importantly, when the binocular DCNVA comparison was subjected to the same covariate-adjusted ANCOVA approach applied to monocular outcomes (Table 5), the between-group difference did not retain statistical significance after adjustment for either ACD (adjusted difference +0.067 logMAR; *p* = 0.071) or IOL power (adjusted difference +0.063 logMAR; *p* = 0.096). This attenuation after covariate adjustment further supports the interpretation that the unadjusted finding is likely confounded by the baseline biometric imbalances between groups rather than reflecting a genuine lens-specific effect. This finding therefore warrants further investigation in prospective, adequately powered, randomized studies with balanced biometric profiles, and requires dedicated optical bench and psychophysical investigation. It should also be noted that, despite ANCOVA adjustment, the significant imbalance in IOL power (mean difference 1.57 D; *p* = 0.026) and the borderline difference in ACD (Cohen’s d = 0.60; *p* = 0.056) may represent residual sources of confounding. Our single-covariate ANCOVA models, constrained by the available sample size, could not simultaneously control for both covariates, and their combined influence on binocular DCNVA cannot be fully excluded. The unadjusted binocular near vision observation of Evolux™ should therefore be regarded as hypothesis-generating pending replication in a prospective, adequately powered, randomized design. The anterior surface profilometry of the Evolux™ IOL confirmed the presence of concentric zones with alternating spherical aberration signs across the central 4.5 mm optical area, consistent with the manufacturer’s description of its wavefront-engineered non-diffractive design [16,17]. The standard aspheric monofocal profile observed at the periphery aligns with the lens’s intended behaviour under varying pupil diameters. This structural characterisation is purely descriptive and provides a basis for understanding the optical design principles of the Evolux™ optic. It does not permit causal inference between any specific structural feature and the clinical outcomes observed in the present study. Any proposed relationship between the optical profile and the observed clinical findings should be regarded as speculative and hypothesis-generating only; verification would require prospective optical bench studies and in vivo studies with controlled experimental conditions. Given that pupil size might influence the relative contribution of central versus peripheral optical zones, differences in photopic and mesopic pupil diameter between populations may partly explain variability in outcomes across studies. In an optical bench study, Alarcon et al. reported that the TECNIS Eyhance^TM^ showed simulated distance visual acuity comparable to that of standard aspheric monofocal IOLs, with an improvement in intermediate simulated visual acuity that was independent of pupil size across the tested range of 2 to 5 mm [26]. In the present series, no statistically significant between-group differences in pupil diameter were observed under any lighting condition, which partially reduces this confounding factor. Nevertheless, future optical bench and in vivo studies examining how these lenses perform across a range of controlled pupil diameters would be informative. The performance of the TECNIS Eyhance™ IOL in the literature has been compared not only with standard monofocal lenses but also with EDOF designs. When evaluated against EDOF IOLs, Eyhance™ has demonstrated similar intermediate visual acuity with a lower incidence of dysphotopsia, at the cost of reduced near performance [13,14]. The present study did not include an EDOF comparator, but dysphotopsia symptoms were rare in both groups. Median scores were “Never” for all items in both groups, except for pain or burning in the Evolux™ group, which showed a median of “Rarely”, though no between-group difference reached statistical significance. The low dysphotopsia burden is clinically relevant in the context of patient counselling, particularly for individuals who prioritize optical quality and night-vision comfort over maximal spectacle independence. Patient-reported outcomes from the Revised DATE questionnaire were broadly comparable across groups. Overall satisfaction was high in both cohorts (73.7% vs. 84.6%), willingness to select the same lens again was similarly elevated (89.5% vs. 84.6%), and no questionnaire item reached statistical significance. A numerically higher rate of “always” reading glasses use was observed in the TECNIS Eyhance™ group (78.9% vs. 46.2%), consistent with the trend in spherical equivalent, as the slight myopic shift in the Evolux™ group may have provided a modest unaided near vision advantage in daily life, though this difference did not reach significance (MWU *p* = 0.088) and should be interpreted cautiously given the limited sample. Taken together, patient-reported data are broadly consistent with the objective outcome findings and suggest that both lenses were associated with high satisfaction and comparable functional vision in the early postoperative period. However, given the limited sample size, the absence of confirmatory statistical power, and the differential response rates between groups, these data should be interpreted cautiously and do not constitute evidence of clinical equivalence. The differential questionnaire response rate between groups (93% in the Evolux™ group versus 63% in the TECNIS Eyhance™ group) introduces a substantial risk of response bias that materially limits the comparability of patient-reported data. Non-responding patients in the TECNIS Eyhance™ group may differ systematically from respondents in terms of satisfaction, visual experience, or functional outcomes, and the direction of this potential bias cannot be determined from the available data. All conclusions drawn from the questionnaire results should therefore be regarded as preliminary and interpreted with caution, and no inference regarding comparative patient-reported outcomes should be considered robust. The primary limitation of this study is the risk of selection bias arising from its retrospective, non-randomized design combined with the imbalance in sample size between groups (30 versus 14 patients). Because IOL allocation was determined by clinical judgement and institutional lens availability rather than by randomization, systematic differences between the two patient cohorts cannot be excluded. The statistically significant difference in IOL power and the medium-magnitude difference in anterior chamber depth observed at baseline are consistent with this concern and may reflect unmeasured confounding that single-covariate ANCOVA adjustment is unlikely to fully eliminate given the constraints of the study design. These factors limit the validity of all between-group comparisons and must be considered when interpreting every finding reported in this study. Additional limitations include the small total sample size, the resulting limited statistical power, the inability to perform simultaneous adjustment for multiple covariates and the short follow-up. Postoperative assessments were performed at one month after second-eye surgery, a timeframe that is unlikely to capture the full extent of neuroadaptation to the implanted lens design which has been shown to evolve at the cortical level up to three to six months following IOL implantation [27]. Consequently, the functional benefits of both lenses may be underestimated at this early assessment point. Furthermore, the refractive stability of the Evolux™ group, which showed a trend toward a myopic shift, cannot be confirmed at one month; longer follow-up would be required to determine whether this shift persists, resolves, or progresses [16]. This source of variability is particularly relevant for binocular outcomes and patient-reported measures, which are known to be influenced by the cumulative duration of binocular exposure to the implanted lens design. It also precludes assessment of neuroadaptation and could meaningfully influence both objective visual acuity outcomes and patient-reported measures. A further source of variability relates to the interval between first-eye and second-eye surgery, which averaged 30 ± 10 days. This variability may have introduced heterogeneity in binocular neuroadaptation status at the time of assessment, particularly affecting binocular visual acuity outcomes and patient-reported measures. The questionnaire response rate was substantially lower in the TECNIS Eyhance™ group (63%, 19/30 patients) than in the Evolux™ group (93%, 13/14 patients). This differential non-response constitutes a potential source of selection bias: if patients with poorer outcomes or lower satisfaction were less likely to complete the questionnaire in the TECNIS Eyhance™ group, between-group comparisons of patient-reported outcomes may be distorted, potentially inflating apparent equivalence. This limits the interpretation of all DATE questionnaire comparisons, and future studies should incorporate systematic follow-up strategies to minimise missing data. No correction for multiple comparisons was applied. Given the exploratory nature of this analysis and the large number of outcomes tested across monocular, binocular, and patient-reported domains, the risk of type I error is elevated and all findings should be interpreted as hypothesis-generating. No individual result should be considered confirmatory in the absence of adequate prospective replication. From a clinical counselling perspective, both lenses might be suitable choices for patients who prioritize computer use, driving, and intermediate-distance tasks. The unadjusted binocular near vision finding for Evolux™ did not retain significance after covariate adjustment and cannot currently be used as a counselling point; it should therefore be interpreted with caution pending confirmation in adequately powered prospective studies. The comparable dysphotopsia profiles support the use of either lens in patients who are particularly sensitive to optical side effects. Future prospective, randomized trials with larger and balanced cohorts, standardized biometric selection criteria, and longer follow-up are essential to definitively characterise the comparative clinical performance of these two lenses. Studies that systematically examine the effect of pupil size, binocular summation mechanisms, and formula optimization on outcomes with these novel optical profiles would be particularly valuable.

## Figures and Tables

**Table 1:** Demographic and biometric data, TECNIS Eyhance™ and Evolux™ (patient-level; *n* = 30 and *n* = 14, respectively).

Variable	TECNIS Eyhance™ (*n* = 30)	Evolux™ (*n* = 14)	Diff (TECNIS Eyhance™—Evolux™)	95% CI	Cohen’s d	*p*-Value
Age (years)	73.29 ± 6.31	74.50 ± 6.09	−1.21	[−5.35, 2.92]	−0.20	0.552
ACD (mm)	3.15 ± 0.39	2.92 ± 0.33	0.23	[−0.01, 0.46]	0.60	0.056
IOL power (D)	22.16 ± 1.77	20.59 ± 2.13	1.57	[0.21, 2.93]	0.83	0.026 *
AL (mm)	23.15 ± 0.61	22.56 ± 2.51	0.59	[−0.87, 2.05]	0.40	0.399
WTW (mm)	11.90 ± 0.34	11.80 ± 0.32	0.11	[−0.11, 0.32]	0.32	0.320
ΔK (D)	−0.49 ± 0.18	−0.41 ± 0.20	−0.08	[−0.21, 0.05]	−0.41	0.232
Scotopic pupil (mm)	4.25 ± 0.71	4.52 ± 0.90	−0.27	[−0.80, 0.27]	−0.35	0.317
Mesopic pupil (mm)	3.61 ± 0.61	3.83 ± 0.74	−0.22	[−0.64, 0.21]	−0.33	0.307
Photopic pupil (mm)	2.89 ± 0.50	2.90 ± 0.65	−0.01	[−0.41, 0.39]	−0.02	0.962

ACD = anterior chamber depth; AL = axial length; CI = confidence interval; SD = standard deviation; WTW = white-to-white; ΔK = flat K − steep K. Values are mean ± SD. Welch’s *t*-test. * *p* < 0.05. Differences may not equal the subtraction of displayed means due to rounding.

**Table 2:** Postoperative monocular visual acuity and refraction (patient-level).

Variable	TECNIS Eyhance™ (*n* = 30 Patients, 60 Eyes)	Evolux™ (*n* = 14 Patients, 28 Eyes)	Diff (TECNIS Eyhance—Evolux™)	95% CI	Cohen’s d	*p*-Value	TOST *p*
SE (D)	0.032 ± 0.275	−0.107 ± 0.258	0.139	[−0.035, 0.314]	0.517	0.113	0.103
mono-UDVA (logMAR) ^††^	0.003 ± 0.080	−0.018 ± 0.089	0.021	[−0.037, 0.079]	0.256	0.455	0.005 †
mono-CDVA (logMAR) ^††^	−0.030 ± 0.048	−0.064 ± 0.072	0.034	[−0.010, 0.079]	0.604	0.122	0.003 †
mono-UIVA (logMAR) ^††^	0.198 ± 0.119	0.232 ± 0.095	−0.034	[−0.102, 0.034]	−0.302	0.320	0.028 †
mono-DCIVA (logMAR) ^††^	0.168 ± 0.134	0.200 ± 0.071	−0.032	[−0.094, 0.031]	−0.268	0.311	0.016 †
mono-DCNVA (logMAR) ^††^	0.255 ± 0.118	0.325 ± 0.112	−0.070	[−0.146, 0.006]	−0.601	0.069	0.212
mono-CNVA (logMAR) ^††^	0.023 ± 0.054	0.011 ± 0.029	0.013	[−0.013, 0.038]	0.266	0.318	<0.001 †
NVC (D) ^††^	1.396 ± 0.574	1.429 ± 0.432	−0.033	[−0.350, 0.285]	−0.061	0.835	0.003 †

CDVA = corrected distance visual acuity; CNVA = corrected near visual acuity; CI = confidence interval; DCIVA = distance-corrected intermediate visual acuity; DCNVA = distance-corrected near visual acuity; mono = monocular; NVC = near vision correction; SE = spherical equivalent; TOST = two one-sided tests; UDVA = uncorrected distance visual acuity; UIVA = uncorrected intermediate visual acuity. † TOST equivalence demonstrated (pTOST < 0.05; margin ± 0.1 logMAR for visual acuity, ± 0.25 D for SE, ± 0.5 D for NVC). Differences may not equal the subtraction of displayed means due to rounding. †† Values represent patient-level means (average of right and left eye). TECNIS Eyhance™: *n* = 30 patients (60 eyes); Evolux™: *n* = 14 patients (28 eyes).

**Table 3:** Postoperative binocular visual acuity (patient-level).

Variable	TECNIS Eyhance™ (*n* = 30)	Evolux™ (*n* = 14)	Diff (TECNIS Eyhance—Evolux™)	95% CI	Cohen’s d	*p*-Value	TOST *p*
bino-UDVA (logMAR)	−0.030 ± 0.070	−0.071 ± 0.099	+0.041	[−0.020, +0.103]	0.515	0.176	0.031 †
bino-CDVA (logMAR)	−0.070 ± 0.053	−0.021 ± 0.080	−0.049	[−0.098, +0.001]	−0.771	0.053	0.021 †
bino-UIVA (logMAR)	0.170 ± 0.121	0.179 ± 0.105	−0.009	[−0.082, 0.064]	−0.074	0.812	0.008 †
bino-DCIVA (logMAR)	0.130 ± 0.115	0.150 ± 0.076	−0.020	[−0.079, 0.039]	−0.192	0.498	0.005 †
bino-DCNVA (logMAR)	0.233 ± 0.112	0.164 ± 0.084	0.069	[0.007, 0.131]	0.661	0.030 *	0.158
bino-CNVA (logMAR)	0.023 ± 0.050	0.007 ± 0.027	0.016	[−0.007, 0.040]	0.364	0.172	<0.001 †

bino = binocular; CDVA = corrected distance visual acuity; CNVA = corrected near visual acuity; DCIVA = distance-corrected intermediate visual acuity; DCNVA = distance-corrected near visual acuity; TOST = two one-sided tests; UDVA = uncorrected distance visual acuity; UIVA = uncorrected intermediate visual acuity. † TOST equivalence demonstrated. * *p* < 0.05 Welch’s t.

**Table 4:** ANCOVA, monocular outcomes adjusted for ACD and IOL power (patient-level).

Outcome	Covariate	Adj. Diff. (TECNIS Eyhance™—Evolux™)	Std. Error	*p*-Value
SE	ACD	0.107	0.090	0.243
SE	IOL power	0.139	0.095	0.150
mono-UDVA ^††^	ACD	0.036	0.027	0.181
mono-UDVA ^††^	IOL power	0.021	0.029	0.473
mono-CDVA ^††^	ACD	0.043	0.019	0.027 *
mono-CDVA ^††^	IOL power	0.038	0.020	0.065
mono-UIVA ^††^	ACD	−0.016	0.037	0.671
mono-UIVA ^††^	IOL power	−0.044	0.039	0.269
mono-DCIVA ^††^	ACD	−0.018	0.040	0.658
mono-DCIVA ^††^	IOL power	−0.039	0.042	0.350
mono-DCNVA ^††^	ACD	−0.070	0.040	0.086
mono-DCNVA ^††^	IOL power	−0.080	0.041	0.057
mono-CNVA ^††^	ACD	0.012	0.016	0.455
mono-CNVA ^††^	IOL power	0.015	0.017	0.375

ACD = anterior chamber depth; Adj. diff. = adjusted difference (TECNIS Eyhance™ − Evolux™); CDVA = corrected distance visual acuity; CNVA = corrected near visual acuity; DCIVA = distance-corrected intermediate visual acuity; DCNVA = distance-corrected near visual acuity; logMAR = logarithm of the minimum angle of resolution; mono = monocular; SE = spherical equivalent; UDVA = uncorrected distance visual acuity; UIVA = uncorrected intermediate visual acuity. For visual acuity outcomes (logMAR): positive = TECNIS Eyhance™ worse. For spherical equivalent (D): positive = TECNIS Eyhance™ closer to emmetropia (better refractive outcome). * *p* < 0.05. †† Values represent patient-level means (average of right and left eye). TECNIS Eyhance™: *n* = 30 patients (60 eyes); Evolux™: *n* = 14 patients (28 eyes).

**Table 5:** ANCOVA, binocular outcomes adjusted for ACD and IOL power (patient-level).

Outcome	Covariate	Adj. Diff. (TECNIS Eyhance™—Evolux™)	Std. Error	*p*-Value
bino-UDVA	ACD	+0.064	0.025	0.015 *
bino-UDVA	IOL power	+0.038	0.028	0.188
bino-CDVA	ACD	−0.016	0.020	0.428
bino-CDVA	IOL power	−0.045	0.022	0.047 *
bino-UIVA	ACD	+0.007	0.039	0.866
bino-UIVA	IOL power	−0.017	0.041	0.673
bino-DCIVA	ACD	−0.009	0.036	0.804
bino-DCIVA	IOL power	−0.028	0.037	0.451
bino-DCNVA	ACD	+0.067	0.036	0.071
bino-DCNVA	IOL power	+0.063	0.037	0.096
bino-CNVA	ACD	+0.023	0.015	0.127
bino-CNVA	IOL power	+0.009	0.015	0.556

ACD = anterior chamber depth; Adj. diff. = adjusted difference (TECNIS Eyhance™ − Evolux™); CDVA = corrected distance visual acuity; CNVA = corrected near visual acuity; DCIVA = distance-corrected intermediate visual acuity; DCNVA = distance-corrected near visual acuity; UDVA = uncorrected distance visual acuity; UIVA = uncorrected intermediate visual acuity. For all visual acuity outcomes (logMAR): positive = TECNIS Eyhance™ worse (higher logMAR). * *p* < 0.05. TECNIS Eyhance™: *n* = 30 patients; Evolux™: *n* = 14 patients. Covariates represent patient-level means (average of right and left eye).

**Table 6:** DATE questionnaire, functional activities without glasses.

Item	TECNIS Eyhance™ (*n* = 19) Yes/Partly/No	Evolux™ (*n* = 13) Yes/Partly/No	RD (pp)	Fisher *p*	MWU *p*
*Satisfaction & recommendation*					
Perform daily routine activities? §	19 (100%)/0/0	13 (100%)/0/0	0.0	-	-
Happy with outcome?	14 (73.7%)/5 (26.3%)/0	11 (84.6%)/1 (7.7%)/1 (7.7%)	−10.9	0.671	0.575
Select same lens again?	17 (89.5%)/2 (10.5%)/0	11 (84.6%)/2 (15.4%)/0	+4.9	1.000	0.713
Would recommend?	17 (89.5%)/2 (10.5%)/-	11 (84.6%)/2 (15.4%)/-	+4.9	1.000	-
*Glass-free functional activities*					
Watch TV	16 (84.2%)/1 (5.3%)/2 (10.5%)	13 (100%)/0/0	−15.8	0.253	0.149
Drive daytime	13/19 drive; all Yes	8/13 drive; all Yes	-	-	-
Drive at night	12/19 drive; all Yes	8/13 drive; all Yes	-	-	-
Shopping in supermarket	10 (52.6%)/7 (36.8%)/2 (10.5%)	4 (30.8%)/6 (46.2%)/3 (23.1%)	+21.9	0.289	0.196
Work at home/garden	17 (89.5%)/1 (5.3%)/1 (5.3%)	10 (76.9%)/1 (7.7%)/2 (15.4%)	+12.6	0.375	0.346
Work at PC †	5 (26.3%)/2 (10.5%)/5 (26.3%)	3 (23.1%)/1 (7.7%)/3 (23.1%)	+3.2	1.000	1.000
Read newspapers ‡	3 (15.8%)/4 (21.1%)/12 (63.2%)	2 (15.4%)/4 (30.8%)/6 (46.2%)	+0.4	1.000	0.567
Read a book ‡	2 (10.5%)/3 (15.8%)/14 (73.7%)	0 (0%)/3 (23.1%)/9 (69.2%)	+10.5	0.510	0.832
Precision work (e.g., sewing)	2 (10.5%)/1 (5.3%)/16 (84.2%)	2 (15.4%)/0/11 (84.6%)	−4.9	1.000	1.000
Completed questionnaire with glasses ¶	14 (73.7%)/5 (26.3%)	7 (53.8%)/6 (46.2%)	+20.0	-	-

RD = risk difference (TECNIS Eyhance™ − Evolux™) in percentage points for “Yes” responses. Fisher’s exact test (Yes vs. Partly + No). MWU = Mann–Whitney U on ordered scores. † Respondents who answered ‘not applicable’ were excluded from the denominator of statistical tests; proportions shown use the total group *n*. ‡ One Evolux™ patient answered not applicable; proportion uses total group *n* = 13. § All respondents answered Yes; statistical testing not applicable. ¶ With glasses/Without glasses (*n*(%)/*n*(%)); formal statistical testing not performed.

**Table 7:** DATE questionnaire, dysphotopsia and glasses use.

Item	TECNIS Eyhance™ (*n* = 19) Median (Range)	Evolux™ (*n* = 13) Median (Range)	MWU	*p*-Value
*Dysphotopsia*				
Glare sensitivity—daytime	Never (0–3)	Never (0)	130.0	0.445
Glare sensitivity—night	Never (0–4)	Never (0–3)	119.0	0.789
Pain or burning	Never (0–2)	Rarely (0–2)	97.5	0.266
Halos	Never (0–3)	Never (0–1)	127.5	0.791
Double images	Never (0–3)	Never (0–2)	118.0	0.791
Problems in bright light	Never (0–4)	Never (0–3)	120.5	0.912
Problems in normal light	Never (0)	Never (0–2)	114.0	0.252
Problems in dim/low light	Never (0–4)	Never (0–2)	139.0	0.426
*Glasses use (Never → Always)*				
Reading glasses	Always (78.9% always)	Always (46.2% always)	161.5	0.088
Glasses for intermediate	Mixed (31.6% always)	Mixed (23.1% always)	138.0	0.580
Glasses for distance	Never (84.2% never)	Never (100% never)	143.0	0.149

Ordinal scale: Never = 0, Rarely = 1, Sometimes = 2, Frequently = 3, Always = 4. Mann–Whitney U test. Median and min–max range of ordinal scores are shown.

## Data Availability

The original contributions presented in this study are included in the article. Further inquiries can be directed to the corresponding author.

## Abbreviations

The following abbreviations are used in this manuscript:

**Abbreviation**	**Definition**
ACD	anterior chamber depth
AL	axial length
ANCOVA	analysis of covariance
CDVA	corrected distance visual acuity
CI	confidence interval
CNVA	corrected near visual acuity
DATE	Revised Heidelberg Daily Task Evaluation
DCIVA	distance-corrected intermediate visual acuity
DCNVA	distance-corrected near visual acuity
EDOF	extended depth of focus
ETDRS	Early Treatment Diabetic Retinopathy Study
IOL	intraocular lens
IOP	intraocular pressure
MWU	Mann–Whitney U
NVC	near vision correction
OCT	optical coherence tomography
pp	percentage points
RD	risk difference
SD	standard deviation
SE	spherical equivalent
TOST	two one-sided tests
UDVA	uncorrected distance visual acuity
UIVA	uncorrected intermediate visual acuity
UV	ultraviolet
WTW	white-to-white
ΔK	corneal curvature gradient (flat K minus steep K)
ΔZ	surface height deviation
